# A CRE/AP-1-Like Motif Is Essential for Induced Syncytin-2 Expression and Fusion in Human Trophoblast-Like Model

**DOI:** 10.1371/journal.pone.0121468

**Published:** 2015-03-17

**Authors:** Chirine Toufaily, Adjimon Gatien Lokossou, Amandine Vargas, Éric Rassart, Benoit Barbeau

**Affiliations:** Département des Sciences Biologiques and Centre de recherche BioMed, Université du Québec à Montréal, Montréal, Canada; North Carolina State University, UNITED STATES

## Abstract

Syncytin-2 is encoded by the envelope gene of Endogenous Retrovirus-FRD (ERVFRD-1) and plays a critical role in fusion of placental trophoblasts leading to the formation of the multinucleated syncytiotrophoblast. Its expression is consequently regulated in a strict manner. In the present study, we have identified a forskolin-responsive region located between positions -300 to -150 in the Syncytin-2 promoter region. This 150 bp region in the context of a minimal promoter mediated an 80-fold induction of promoter activity following forskolin stimulation. EMSA analyses with competition experiments with nuclear extracts from forskolin-stimulated BeWo cells demonstrated that the -211 to -177 region specifically bound two forskolin-induced complexes, one of them containing a CRE/AP-1-like motif. Site-directed mutagenesis of the CRE/AP-1 binding site in the context of the Syncytin-2 promoter or a heterologous promoter showed that this motif was mostly essential for forskolin-induced promoter activity. Transfection experiments with dominant negative mutants and constitutively activated CREB expression vectors in addition to Chromatin Immunoprecipitation suggested that a CREB family member, CREB2 was binding and acting through the CRE/AP-1 motif. We further demonstrated the binding of JunD to this same motif. Similar to forskolin and soluble cAMP, CREB2 and JunD overexpression induced Syncytin-2 promoter activity in a CRE/AP-1-dependent manner and Syncytin-2 expression. In addition, BeWo cell fusion was induced by both CREB2 and JunD overexpression, while being repressed following silencing of either gene. These results thereby demonstrate that induced expression of Syncytin-2 is highly dependent on the interaction of bZIP-containing transcription factors to a CRE/AP-1 motif and that this element is important for the regulation of Syncytin-2 expression, which results in the formation of the peripheral syncytiotrophoblast layer.

## Introduction

During pregnancy, placental development involves the differentiation of placental trophoblasts into two different pathways, i.e the extravillous cytotrophoblast and the villous cytotrophoblast. Villous cytotrophoblasts possess the ability to fuse with adjacent cells and thereby lead to the formation of the peripheral multinucleated syncytiotrophoblast layer. This layer is essential for proper placental development and for the maintenance of normal pregnancy and fetus development. It is responsible for gas exchange between mother and fetus, feto-maternal immunotolerance, nutrient transport and hormone production [1–4]. Failure of syncytiotrophoblast formation is associated with different complications, such as pre-eclampsia, one of the most important cause of maternal morbidity and mortality, preterm birth, perinatal death, and intrauterine growth restriction [5]. Maintenance of the syncytiotrophoblast structure relies on newly fused cytotrophoblasts, a process that is regulated by different transcription factors, growth factors, cytokines, protein kinases and fusogenic proteins such as former envelope (Env) glycoproteins of human Endogenous Retroviruses (ERVs) Syncytin-1 of ERVW-1 and Syncytin-2 of ERVFRD-1.

Human ERVs represent 8% of our genome and are the remnant of exogenous infection that has occurred many million years ago. The human placenta expresses a large number of retroviral elements and their role in the development of this organ seems essential for trophoblast differentiation and syncytiotrophoblast formation. One former Env gene, Syncytin-1 expressed from a deficient proviral DNA, known as ERVW-1, has maintained its fusogenic activity, and its role in trophoblast fusion has been confirmed in early studies [6–10]. The implication of Syncytin-1 in the normal development of the placenta is mediated by its interaction with its receptors ASCT1 (also known as SLC1A4) and mainly ASCT2 (SLC1A5) [8, 11]. Furthermore, Syncytin-1 expression is downregulated in placentas and primary cytotrophoblasts from patients with pre-eclampsia symptoms [12–19], while no such downregulation has been observed for ASCT2 [20]. A recent study has attributed reduced Syncytin-1 expression in pre-eclamptic placenta to hypermethylation of the promoter region [21]. GCM1 (Glial Cells Missing factor 1) is an essential transcription factor for the expression of Syncytin-1 and is dependent on MAPK14 (also known as p38) phosphorylation [22, 23]. Other transcription factors such as SP1, GATA2 and GATA3 were also found to significantly stimulate Syncytin-1 promoter activity [24].

Syncytin-2 is expressed from ERV-1 FRD proviral DNA and has also been implicated in the development of the placenta [25–29]. Indeed, this ERV envelope protein induces fusion of primary cytotrophoblasts as well as choriocarcinoma-derived BeWo cells, which fuses after stimulation with forskolin [30]. Syncytin-2 interacts with a receptor identified as MFSD2a (Major Facilitator Superfamily Domain 2a), a potential member of the carbohydrate transporter family [31] and we have previously demonstrated that this receptor was indeed important for BeWo fusion [32]. Like Syncytin-1, Syncytin-2 is also upregulated following induced increase in cAMP levels in BeWo cells [30]. Furthermore, similarly to Syncytin-1, Syncytin-2 expression is downregulated in pre-eclamptic placentas and inversely correlate with symptom severity [13, 15, 18, 19]. Syncytin-2 is transcribed as a typical singly spliced *env* mRNA starting in the 5’ LTR region and terminating in the 3’ LTR. Transcripts initiate from a single transcription initiation site, which is located downstream of a putative TATA box [30]. One study has demonstrated that the GCM1 transcription factor could be one factor regulating Syncytin-2 as well as its cognate receptor MFSD2a [33].

The implication and expression of GCM1 in BeWo cell fusion has been previously suggested to depend on several transcription factors, such as CREB [34]. This is of particular interest as CREB and CREB2 transcription factors have been associated to the regulation of several trophoblast genes [22, 34, 35]. CREB/ATF family is a large family of transcription factors with a basic-region leucine zipper that bind to a CRE-binding motif. CREB/ATF1 (chromosome 2q34) is very well characterized member and comparison to other members, such as ATF2 (chromosome 2q32), ATF3 (chromosome 9q13), and ATF4 (chromosome 22q13) revealed differences in their structure. CREB/ATF members can act as either homodimers or heterodimers, and are also capable of interacting with members of fos and jun families thereby affecting binding affinity toward different binding sites [36, 37].

Although Syncytin-2 is known for its important role in trophoblast fusion, the mechanism supporting Syncytin-2 regulation remains poorly understood. In the present study, we have used the BeWo cell line model and primary villous cytotrophoblasts isolated from term placenta to study the regulation of Syncytin-2 expression at the promoter level. Our results have led to the identification a CRE/AP-1 motif, which is essential for forskolin-induced expression of Syncytin-2. We demonstrate that bZIP transcription factors, such as CREB2 (ATF4) and JunD interact with this region and transactivate promoter activity as well as BeWo cell fusion. Furthermore, mutations of this CRE/AP-1 motif dramatically decreased Syncytin-2 promoter activity. Our results are thus highlighting a novel element involved in the restricted regulation of Syncytin-2 expression.

## Materials and Methods

### Ethics statement

The UQAM ethic committee has approved the current research project, which is in accordance with the established guidelines of the ethic committee of St-Luc Hospital of the Centre Hospitalier Universitaire de Montréal (Montréal, Canada). All participating women have signed an informed consent form.

### Cell culture

The BeWo choriocarcinoma cell line was obtained from the American Type Culture Collection (Manassas, CA) and maintained in Ham’s F-12 medium supplemented with 10% fetal bovine serum (FBS) (Sigma-Aldrich, Oakville Canada) at 37°C in a 5% CO_2_ atmosphere [38]. For culture of primary cytotrophoblasts, placentas (from 35 to 41 gestational weeks) were obtained after spontaneous vaginal delivery in accordance with the established guidelines of the ethic committee of St-Luc Hospital of the Centre Hospitalier Universitaire de Montréal (Montréal, Canada) and Université du Québec à Montréal (Montréal, Canada). All participating women had signed an informed consent form. For isolation of villous cytotrophoblasts, placental villi were first cut, thoroughly washed and digested in Hanks balanced salt solution containing trypsin (from 9.6 × 10^5^ to 1.8 × 10^6^ U) and DNase I (D5025, Sigma). Dispersed cells were layered on top of a discontinuous Percoll gradient and centrifuged at 507*g*. Intermediate layers containing cytotrophoblasts were collected and washed extensively. Cells were seeded at a density of 1.5 × 10^6^ cells per well in Dulbecco modified eagle medium (high glucose) (Wisent), 2 mM glutamine, 10% FBS and penicillin/streptomycin/neomycin (Invitrogen Canada Inc., Burlington, Canada) and cultured for a maximum of 4 days. The purity of each cytotrophoblast preparation was evaluated by flow cytometry using FITC-conjugated monoclonal antibody against cytokeratin 7 (Millipore, Mississauga, Canada), a specific marker of trophoblasts. Experiments with primary cytotrophoblasts were conducted in triplicates with three different placenta donors.

### Plasmids

pGL3/2600 was generated by PCR amplification of a 2600 bp region flanking the previously described transcription initiation site of the Syncytin-2 gene [30] using 293T genomic DNA and the following primers (5’-CACTAGGGAAGGTATCCGAGTC-3’; 5’-TGCAATGAGGCAAAAGCTAA-3’). The amplified Syncytin-2 promoter region was confirmed by sequencing. A series of 5’ deletion mutants of the Syncytin-2 promoter was also generated by PCR with a common reverse primer 5’-ATCCGAGTAACTGCACCATAAGCTT-3’ and a series of forward primers (Table 1). PCR-amplified fragments were digested with SacI and HindIII (sites present at the extremity of forward and reverse primers, respectively) and inserted into similarly digested pGL3Basic (Promega, Madison, WI). For pGL2-TATA-150, the-300/-150 bp region was amplified from pGL3/2600 using primers 5’-ATACCCGGGGCAACTTCCTGATAAGATC-3’ (forward) and 5’-GGAGGTACCTTTACTGAAGTTGTGCGCC-3’ (reverse). DNA fragments were digested with SmaI and KpnI (sites present at the extremity of forward and reverse primers, respectively) and cloned into the previously described pGL2-TATA-HTLV-1 construct [39] after removal of the HTLV-1 promoter fragment by SmaI and KpnI. An empty control vector was generated after excision of the HTLV-1 fragment and self-ligation (pGL2-TATA). Expression vectors for constitutively activated CREB (CREB (Y/F)) and a CREB dominant negative (KCREB) along with their control empty vectors were provided by Dr. M. Montminy (Salk Institutes for Biological Sciences, La Jolla CA) and Dr. R.H. Goodman (Vollum Institute for Advanced Biochemical, Research, Portland, OR), respectively [40, 41]. Vector expressing CREB2 (pCIneo-CREB2) and the control empty vector, pCIneo as well as expression vectors for the various Jun family members were kindly provided by Dr. J.M. Mesnard (Université Montpellier 1, Montpellier, France) [42]. The pRc-Actin-lacZ vector contains the β-galactosidase gene under the control of the β-actin promoter.

**Table 1:** Oligonucleotides used for construction of deleted mutants and site-directed mutagenesis of the Syncytin-2 promoter.

2600 pb	5'-GAGCTCTGCAAGAGGCAAAGCTAAA-3'
600 pb	5'-GAGACTCAGCGAGGCTGTCTCCAAAA-3'
450 pb	5'-GAGACTCTGATGATCAGGCGGTTGTTA-3'
300 pb	5'-GAGACTCCAACTTCCTGATAAGATC-3'
150 pb	5'-GAGACTCGTAAACAAATGCACATGTGG-3'
100 pb	5'-GAGACTCAGCCCACCCCAAGGAAAA-3'
50 pb	5'-GAGACTCCGGAACCATGCCTGTA-3'
M1 forward	5'-AATTTTGATAAGGGAAAAATGTCTCAAG-3'
M1 reverse	5'-GTGTTCCTAGAGGAAGGTCAC-3'
M5 forward	5'-TTCTTAAAGGGAAAAATGTCTCAAGA-3'
M5 reverse	5'-CAGGTGTTCCTAGAGGAAGG-3'
M6 forward	5'-TTCCAGGGAAAAATGTCTCAAGAAA-3'
M6 reverse	5'-AGTCAGGTGTTCCTAGAGGAA-3'

### Site directed mutagenesis

Mutations in the putative CRE/AP-1-like motif and GATA-binding site were generated in both pGL3/600 and pGL2-TATA-150 vectors by reverse PCR using High fidelity Phusion polymerase (New England Biolabs, Pickering, Canada) following manufacturer’s instructions. Oligonucleotides used to generate these mutations by PCR amplification are indicated in Table 1 as M1, M5 and M6.

### Transfection and measurement of luciferase activity

BeWo cells (2×10^5^) were co-transfected with wild-type or deletion/mutated promoter constructs (vs. control vector) (200 ng) and pRc-Actin-LacZ (200 ng) with the FuGENE 6 transfection reagent according to manufacturer's instructions (Roche Diagnostics, Indianapolis, IN). Twenty-four hours posttransfection, cells were treated with 50 μM forskolin (or equivalent DMSO concentration) or 150 μM SP-cAMP (vs. the inactive RP-cAMP control compound) (BioLynx, Brockville, Canada) for 12 h. Alternatively, cells were co-transfected for 48 h with promoter constructs (200 ng) along with different expression vectors of CREB or Jun (200 ng) and pRc-Actin-LacZ (200 ng). Primary trophoblasts (1.5 × 10^6^) were microporated with a MP-100 device (Digital Bio, Montreal, Qc) with a reporter construct (0.4 μg) and pRcActin-LacZ (0.1 μg) for normalization. For all experiments, cells were lysed in a 1X lysis buffer (25 mM Tris phosphate, pH 7.8, 2 mM DTT, 1% Triton X-100, 10% glycerol). Luciferase activity was determined as follows. After a freeze/thaw cycle, 25 μl of cellular extracts were transferred to a 96-well luminometer plate and luciferase activity was quantified on a Dynex MLX microplate luminometer (MLX; Dynex Technologies, Chantilly, VA) following a single injection of a luciferase buffer [20 mM tricine, 1.07 mM (MgCO_3_)_4_·Mg(OH)_2_·5H_2_O, 2.67 mM MgSO_4_, 0.1 mM EDTA (ethylene diamine tetraacetic acid), 220 μM coenzyme A, 4.7 μM D-luciferin potassium salt, 530 μM ATP, 33.3 mM DTT]. β-galactosidase activity was measured using the Galacto-Light kit (Applied Biosystems, Bedford, MA) according to manufacturer's instructions. Luciferase activity was calculated in terms of relative light units and represents the mean ± S.E.M of three or more transfected samples normalized for β-galactosidase activity.

### Preparation of nuclear extracts and EMSA

BeWo cells were either left untreated or treated with 50 μM forskolin for 15 min, while primary villous trophoblasts were cultured from 24 to 96 h. Nuclear proteins from BeWo cells of primary trophoblasts were next isolated using the NE-PER nuclear and cytoplasmic extraction reagents according to manufacturer’s instructions (Thermo Fisher Scientific Inc., Rockford, IL) and electrophoretic mobility shift assay was then performed. Single stranded oligonucleotides (50 ng) were 5'-end-labeled using 1 U of T4 polynucleotide kinase and (γ-^32^P) ATP at 37°C. Labeled oligonucleotides were then annealed with 200 ng of their corresponding complementary oligonucleotide. Nuclear extracts (10 μg) were incubated with 0.8 ng of labeled double stranded oligonucleotide at room temperature. The DNA-protein complex was then run on a 5% non-denaturating polyacrylamide gel in a 0.5X TBE buffer, which was then exposed on a Kodak X-Omat film (Perkin Elmer, Rochester, NY). For competition experiments, a 100-fold molar excess of unlabeled oligonucleotides was added to the reaction mixture before incubation with labeled probes.

### Chromatin Immunoprecipitation assays

BeWo cells (0.5×10^6^) were either left untreated or treated with 50 μM forskolin for 15 min. Cells were trypsinized, washed with PBS 1X and resuspended in 500 μl PBS. Cross-linking and immunoprecipitation were conducted using 2 μg of polyclonal antibodies against CREB1, CREB2 or JunD (Santa Cruz Biotechnology, Inc. Santa Cruz, CA) and the M-Fast Chromatin immunoprecipitation Kit according to manufacturer's instructions (ZmTech scientifique, Montreal, Canada). Anti-Histone 3 (H3) and rabbit anti-IgG were used as positive and negative controls, respectively. Immunoprecipitated chromatin was analyzed using primers specific for the Syncytin-2 promoter region (5’-ATACCCGGGGCAACTTCCTGATAAGATC-3’ (forward) and 5’-GGAGGTACCTTTACTGAAGTTGTGCGCC-3’ (reverse)).

### RNA isolation and RT-PCR analyses

Total RNA was isolated from BeWo cells transfected with the CREB (Y/F) expression vector (vs. the control empty vector) (500 ng) and untreated (DMSO) vs. forskolin-treated BeWo cells at different time points using the Rneasy Plus mini Kit (Qiagen, Mississauga, Canada). Prior to RT, total RNA was treated with Turbo RNAse-Free DNAse (Ambion, Austin, TX) for 5 min at 70°C. RNA (1 μg) was then incubated in the presence of oligo(dT) (25 ng/μl), 10 mM DTT, 100 μM dNTP (deoxynucleotide triphosphate), SuperScript reverse transcriptase (10 U) (Invitrogen Canada Inc), and SUPERase-In (20 U) at 42°C for 50 min. Aliquots from the RT reactions were then PCR-amplified in the presence of 2 U Vent DNA polymerase (New England Biolabs, Pickering, Canada), 1× ThermoPol buffer, 100 μM dNTP, and 15 μM of each primer. PCR amplification was performed using Syncytin-2-specific primers 5’-CCTTCACTAGCAGCCTACCG-3’ (forward) and 5’-GCTGTCCCTGGTGTTTCAGT-3’ (reverse); CREB2-specific primers 5’-GTGTCATCCAACGTGGTCAG-3’ (forward) and 5’-CCAACAACAGCAAGGAGGAT-3’ (reverse); and JunD-specific primers 5’-GTGCCCAGGAACTCAGAGAG-3’ (forward) and 5’-CCAACGTTTGTTCCCGAGTA-3’ (reverse). As a control, β-actin mRNA were similarly amplified using primers 5’-CGTGACATTAAGGAGAAGCTG-3’ (forward) and 5’-CTCAGGAGGAGCAATGATCTT-3’ (reverse). Densitometric analyses of band intensities for JunD and CREB2 signals were normalized with corresponding β-actin mRNA levels.

### Western blot analyses

Cellular **e**xtracts (20 μg) were migrated in a 12% SDS-PAGE and transferred on a PVDF membrane (Millipore). Membranes were blocked with 5% powder milk and incubated with anti-JunD (1 μg/ml), anti-CREB2 (1 μg/ml), anti-Syncytin-2 (1/7500) or anti-GAPDH (glyceraldehyde-3-phosphate dehydrogenase) (1/500) antibodies (Santa Cruz Biotechnology Inc., Dallas, TX). After several washes, membranes were further incubated with appropriate horseradish peroxidase (HRP)-conjugated goat anti-rabbit/mouse antibodies (1/10,000) (Cell Signalling, Danvers, MA), and signals were detected using the BM chemiluminescence blotting substrate (Roche Diagnostics). Membranes were scanned with the Fusion FX5 system (Montreal Biotech Inc., Dorval, Canada) and band intensities were determined and normalized with GAPDH with the Quantity One 4.5.0 image acquisition software.

### Cell fusion assay

Cells were either stimulated or not with 50 μM forskolin or 150 μM SP-cAMP (vs. the inactive RP-cAMP control compound) or transfected with 500 ng pCIneo-CREB2, pcDNA3.1/JunD or their corresponding empty vectors. At 48 h post-stimulation or post-transfection, cells were fixed in chilled methanol for 20 min at room temperature and then incubated with PBS containing 2% FBS (v/v) for 30 min to eliminate nonspecific binding. Cells were rinsed with PBS and incubated in the presence of a mouse monoclonal anti-desmoplakin antibody (Sigma-Aldrich, Oakville, Canada, 1/700) in PBS containing 0.2% bovine serum albumin for 1½ h at room temperature, washed 3 times with PBS, and incubated with the Alexa Fluor 488-conjugated goat anti-mouse immunoglobulin IgG (1/1000) for 1 h at room temperature. For nuclear staining, cells were incubated with propidium iodide (50 μg/mL) for an additional 30 min at room temperature, washed 3 times with PBS, and visualized with a confocal laser-scanning microscope (Bio-Rad MRC1024, Hercules, CA). A syncytium was defined as an agglomeration of 2 or more nuclei in the same cytoplasm without intervening surface desmosomal membrane staining. Cellular fusion index was determined as follows: five fields were counted per well and an average was calculated and expressed as the percentage number of nuclei in syncytia. In general, a total of 200 nuclei were counted per field and, thus, nearly 1000 nuclei were counted in each well. Three replicate wells were examined per experimental condition from which a final cellular fusion index (mean ± SEM) is presented and represents the final average of the 3 percentage values.

### siRNA transfection

All siRNAs were synthesized by Invitrogen Canada Inc. (Qiagen): siRNA Ctrl ref SI03650318; siRNA JunD ref SI00075978; siRNA CREB-2 SI03019345. As previously described, BeWo cells plated in 24-well plates (1.5×10^5^ cells) were stimulated with 50 μM forskolin of control DMSO concentration and transfected after 16–24 h of stimulation, using Hiperfect (Qiagen) in the presence of 37.5 ng of siRNA duplexes. At 36 h post-transfection, the specificity of each siRNA was analysed by Western blot and their impact on cell fusion assay was evaluated [30].

### Statistical analysis

All experiments were performed in triplicates. Results are expressed as the mean ± SEM and statistically analyzed using GraphPad prism 5 software with t-test for one group comparison and two-way ANOVA for two or more group analysis with *p< 0.05; ** p< 0.01 and *** p< 0.001 being considered significant.

## Results

### Identification of a forskolin-responsive region in the Syncytin-2 promoter

**Fig 1:**
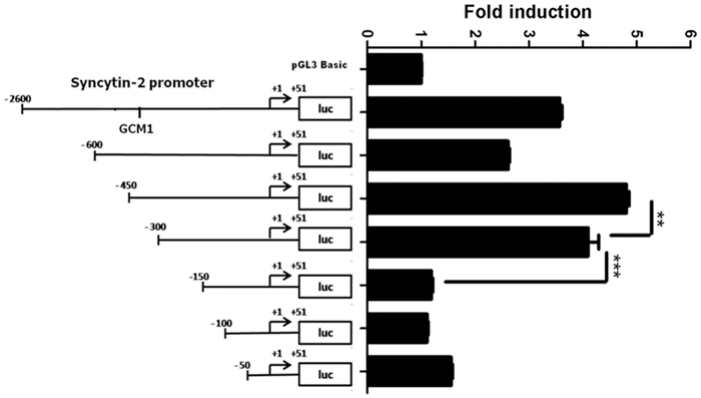
Identification of Syncytin-2 promoter regions responsive to forskolin induction in BeWo cells. The first 2600 bp region positioned upstream of the Syncytin-2 transcription initiation site in addition to the first 51 nucleotide of exon 1 were cloned upstream of the luciferase reporter gene. The resulting vector pGL3/2600 and successive 5’ deletion mutants vs. the control vector pGL3Basic (400 ng) were co-transfected with 200 ng pRcActin-LacZ in BeWo cells. Cells were either left untreated or treated with 50 μM forskolin. At 12 h post treatment, cells were harvested and assayed for luciferase activity in triplicates. Luciferase activities were normalized over β-galactosidase activity as the mean ± S.E.M. from three independently transfected cells in the same experiment. Results are shown as mean of fold induction of treated over untreated cell samples and are representative of four independent experiments (**p < 0.01 *** p < 0.001). The position of the previously identified GCM1-binding site and the transcription initiation site are both shown within the Syncytin-2 promoter in the pGL3/2600 construct.

Forskolin treatment increases cAMP levels, thereby activating both protein kinase A and MAPK14 serine/threonine kinase activities and subsequent expression of the fusogenic genes, Syncytin-1 and Syncytin-2 in the BeWo choriocarcinoma cell line [22]. Former studies had suggested that a GCM1-binding site located at position-428 was important for the induction of the Syncytin-2 promoter [33]. To conduct a more elaborate analysis of the Syncytin-2 promoter, its region was cloned upstream of the luciferase reporter gene and a series of 5’ deletion mutants was generated. Resulting constructs were transfected in BeWo cells, which were then treated with forskolin for 12 h. As shown in Fig. 1, the pGL3/2600 construct showed a 4 fold induction following forskolin treatment. Progressive deletion from-450 to-300 decreased luciferase activity and thereby confirmed a role for the GCM1 transcription factor. However, deletion of the-300 to-150 nucleotide region (pGL3/300) presented a more dramatic reduction in promoter activity, which became comparable to luciferase activity corresponding to pGL3basic-transfected cells.

These results hence suggested that an important region was present in the Syncytin-2 promoter, which did not implicate the GCM1-binding site.

### The-300 to-150 Syncytin-2 promoter region is forskolin-inducible in BeWo cells

**Fig 2:**
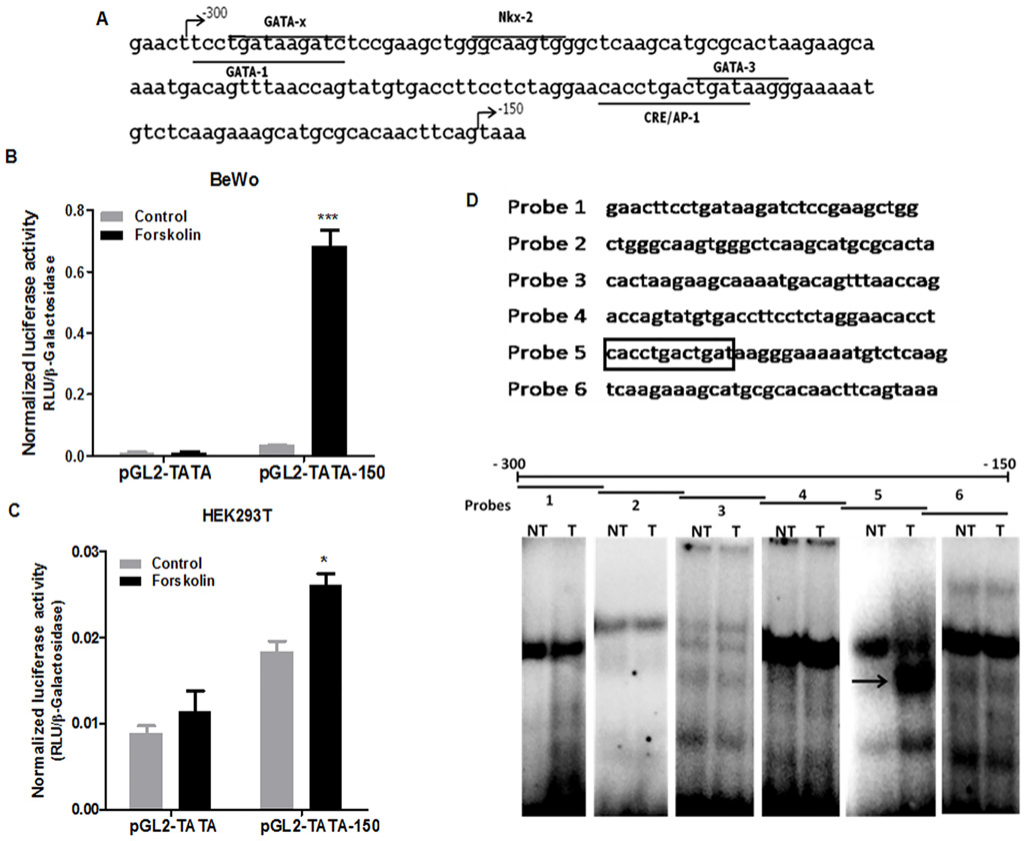
Analysis of the-300 to-150 Syncytin-2 region responsive to forskolin. **(A)** Sequence of the-300 to-150 promoter region of Syncytin-2 in which putative transcription binding sites are underlined. **(B-C)** The pGL2-TATA-150 vector comprising the-300 to-150 Syncytin-2 promoter region and a TATA box upstream of the luciferase reporter gene (vs. the pGL2-TATA control vector) (400 ng) was co-transfected along with 200 ng of pRcActin-LacZ in BeWo **(B)** or 293T cells **(C)**. After transfection, cells were either left untreated (Control) or treated with 50 μM of forskolin for 12 h. Cells were harvested and analyzed for relative luciferase expression. Luciferase activity values were normalized for β-galactosidase activity and are expressed as the mean normalized RLU ± S.E.M. from three independently transfected cells in the same experiment. These results are representative of three independent experiments (*p< 0.05; *** p< 0.001). **(D)** Five different probes covering the-300 to-150 region were used for EMSA. Nuclear extracts (10 μg) from non-treated (NT) or treated (T) BeWo cells were incubated with the different labeled probes covering this 150 nt region. The arrow points to a specific signal observed with probe 5.

To confirm the above results, further analyses were conducted on this-300/-150 region. First, an *in silico* analysis was performed to identify potential transcription factor-binding sites. The analysis revealed that the 150 bp region contained putative consensus elements, such as GATA1-binding sites as well as a CRE/AP-1-like motif (Fig. 2A). To further investigate whether the 150 bp fragment was forskolin-responsive on its own, the region was cloned upstream of a TATA box and the luciferase reporter gene and the resulting vector was transfected in BeWo or 293T cells. After forskolin stimulation, the 150 bp region indeed showed an important increase in luciferase activity (up to 80-fold) in BeWo cells, while no such induction was noted in cells transfected with the control vector (Fig. 2B). In non-trophoblastic 293T cells, transactivation was modest (Fig. 2C) pointing to cell specificity for this induction. To identify specific protein interactions with this region, a series of probes covering the whole sequence located between positions-300 to-150 were generated and incubated with nuclear extracts from non-treated or forskolin-treated BeWo cells. A specific complex was identified with probe 5, only when incubated with nuclear extracts from forskolin-treated BeWo cells (Fig. 2D). Interestingly, probe 5 contained the predicted CRE/AP-1-like motif.

These results indicated that the-300/-150 Syncytin-2 promoter region was strongly responsive to forskolin in BeWo cells and that it displayed binding to a complex in a segment harboring a CRE/AP-1-like motif and a GATA-binding site.

### Binding of specific complexes to the CRE/AP-1 motif

**Fig 3:**
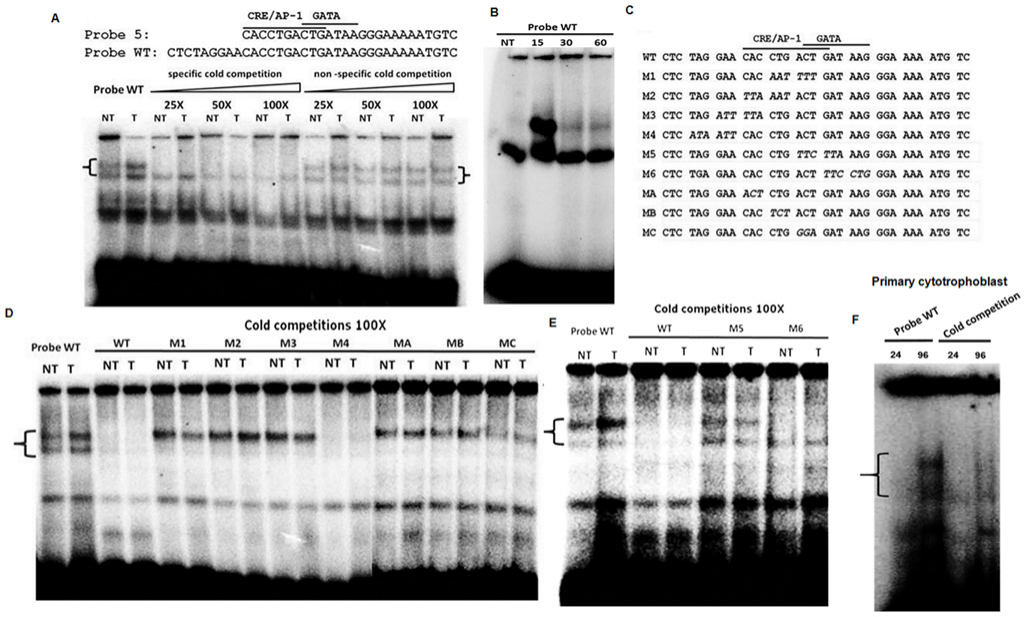
Identification of a CRE/AP-1-like motif and GATA-binding site within the Syncytin-2 promoter. **(A)** Extended probe 5 (termed WT) (from-211 to-177) (indicated in the upper part of the panel) was used for EMSA analyses and highlights the CRE/AP-1-like motif and the potential GATA-binding site in the Syncytin-2 promoter. Nuclear extracts from BeWo cells treated (T) or not (NT) with forskolin for 15 min were pre-incubated with different concentrations of cold specific or non-specific oligonucleotides prior to addition of the labeled WT probe. (**B**) EMSA analyses of nuclear extracts from BeWo cells stimulated with forskolin at different time points and incubated with the WT probe. **(C)** Representation of the various oligonucleotides bearing mutated sequences (nucleotides in italic) used in EMSA analyses. **(D-E)** Nuclear extracts from non-treated (NT) or forskolin-treated (T) BeWo cells were incubated with the WT probe and excess (100X) cold WT or mutated oligonucleotides presented in (**C**). Specific DNA-protein complexes are indicated on left side of panels. (**F**) Nuclear extracts from primary villous cytotrophoblasts cultured for 24 and 96 hours were incubated with the WT probe and with or without excess (100X) cold WT oligonucleotide.

In order to further analyze the complex binding to probe 5, EMSA experiments were performed with a probe further extended at its 5’ end (termed WT) to favor possible binding to the CRE/AP-1 motif, while maintaining the GATA-like binding site (Fig. 3A). Using this new probe, two specific DNA-protein complexes were identified and presented higher intensity in the presence of nuclear extracts from forskolin-treated BeWo cells vs. non-treated cells (Fig. 3A). Addition of cold WT oligonucleotides revealed competition of both complexes, while no similar competition was observed with an unrelated cold oligonucleotide, confirming their specificity (Fig. 3A). A time course experiment revealed that bound complexes were very transient and peaked at 15 minutes, as assessed by analyses of the EMSA results (Fig. 3B). A series of oligonucleotides were next synthesized with mutations in the CRE/AP-1 motif as well as on either side of the motif (Fig. 3C). Competition experiments with unlabeled M1, M2 and M3 oligonucleotides (mutated in the CRE/AP-1-like motif) led to disappearance of the lower complex only, thereby demonstrating the importance of these mutated sequences for the interaction of the upper complex with the motif (Fig. 3D). Mutant M4, bearing modified nucleotides at the 5’ extremity competed with both signals at comparable levels to the wild-type oligonucleotide. Oligonucleotides MA, MB and MC containing three modified nucleotides covering the expected CRE/AP-1 motif confirmed the above competition results (Fig. 3D). Two other mutated oligonucleotides were also tested, M5 and M6, both bearing modified nucleotide 3’ to the CRE/AP-1 motif (Fig. 3E). When mutant M5 was added in cold excess to the labeled WT oligonucleotide and BeWo nuclear extracts, competition was affected for both complexes when compared to competition with the WT oligonucleotide. On the other hand, oligonucleotide M6 could only compete with the upper complex, suggesting that this other complex also interacted specifically with the WT probe but did not require the CRE/AP-1-like motif (Fig. 3E). EMSA experiments were also conducted with nuclear extracts from primary cytotrophoblasts isolated at either day 1 or day 4. DNA-protein interaction was more significant at day 4 compared to day 1 (Fig. 3F), a time point at which both Syncytin-2 expression and cell fusion are at their highest levels [30].

These results thus suggested that the-300/-150 region harbored a CRE/AP-1-like motif with an adjacent GATA-like binding site potentially capable of forming two complexes with greater abundance in stimulated BeWo cells and primary cytotrophoblasts cultured for 96 hours.

### The CRE/AP-1-like motif is critical for forskolin-induced Syncytin-2 promoter activity

**Fig 4:**
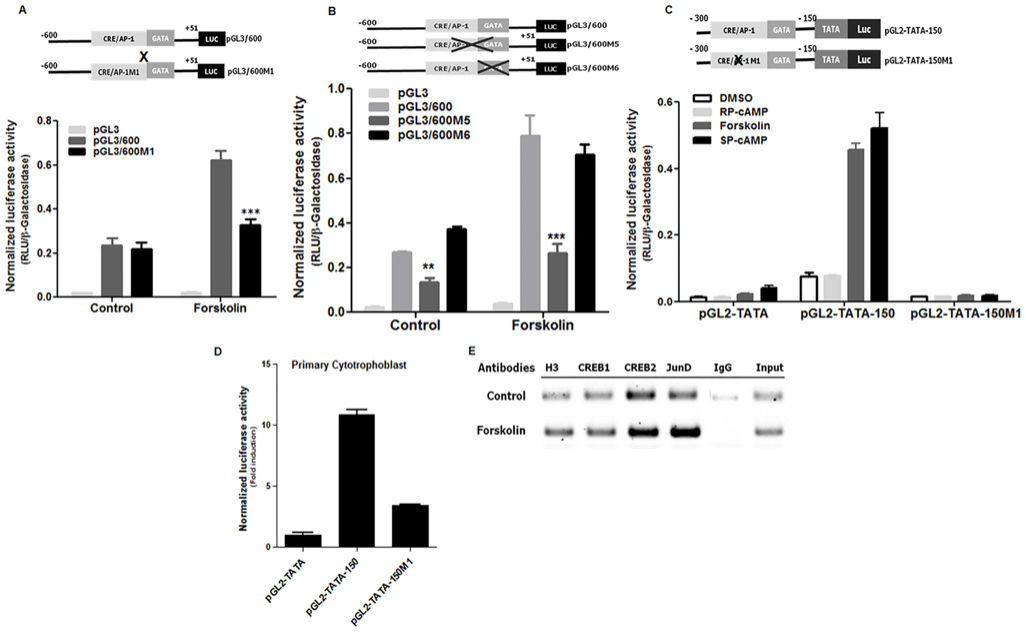
Importance of the CRE/AP-1-like motif in induced Syncytin-2 promoter activity. **(A-C)** BeWo cells were co-transfected with pGL3/600 (400 ng) or equivalent constructs bearing the M1 (**A**), M5 or M6 (**B**) mutant sequences along with pRcActin-LacZ (200 ng). In **C**, BeWo cells were co-transfected with pGL2-TATA-150 or pGL2-TATA-150M1 (vs. the control pGL2-TATA vector) (400 ng) along with pRcActin-LacZ (200 ng). Following transfection, cells were left untreated (Control or DMSO) or treated with forskolin (**A-C**) and RP-cAMP/SP-cAMP (**C**) for 12 h. (**D**) Primary villous cytotrophoblasts were microporated with pGL2-TATA-150 or pGL2-TATA-150M1 (vs. the control pGL2-TATA vector) (400 ng) along with pRcActin-LacZ (100 ng). Cells were then harvested and analyzed for luciferase expression. Luciferase values were normalized for β-galactosidase activity and are expressed as the mean normalized RLU ±S.E.M. from three independently transfected cells in the same experiment. Results in (**D**) are shown as mean of fold induction over pGL2-TATA-transfected cells (set as 1) and are representative of three independent experiments (**p< 0.01; ***p< 0.001). (**E**) Chromatin immunoprecipitation analysis of CREB1, CREB2 and JunD binding to Syncytin-2 promoter region in non-treated or treated BeWo cells was performed with antibodies specific to these factors or against Histone 3 (positive control) or with non-specific IgG antibodies (IgG: negative control). The-300/-150 region of the Syncytin-2 promoter was next PCR amplified on immunoprecipitated DNA. Input DNA was similarly amplified for control.

As mutated M1, M5 and M6 oligonucleotides led to loss of either complexes or both complexes in the above EMSA experiments, we next assessed their impact in the context of the Syncytin-2 promoter. The M1 nucleotidic modification was first introduced in the 600bp promoter construct (pGL3/600M1), which was then transfected in BeWo cells. The M1 mutation significantly reduced forskolin-mediated increase in promoter activity (Fig. 4A). However, this mutation did not alter the basal activity of the Syncytin-2 promoter construct. As our EMSA analyses suggested that a region next to the CRE/AP-1-like motif was also important for the binding of the other complex, we next evaluated if mutation affecting its binding could also alter promoter activity following stimulation of BeWo cells. Two mutants were thereby generated in the context of the 600 bp promoter construct: mutant M5 leading to a reduced affinity for both complexes and mutant M6 solely affecting the binding of the lower complex. When the pGL3/600M5 mutant construct was transfected in BeWo cells, reduced induction of promoter activity was noted, being reminiscent of the M1 mutant construct (Fig. 4B). It should also be noted that basal activity was affected when both CRE/AP-1-like motif and GATA-binding site were mutated (mutant M5). As opposed to these results, the 600bp promoter construct modified at the sequence corresponding to the GATA-like binding site, i.e. the M6 mutation, did not present a significant difference in basal or forskolin-induced activity upon transfection of BeWo cells.

To corroborate the importance of the CRE/AP-1 motif in this induced promoter activity, the M1 mutation was also introduced into the 150 bp fragment positioned upstream to the TATA box and the luciferase reporter gene (pGL2-TATA-150). Mutation within the CRE/AP-1-like motif strongly hampered forskolin-induced luciferase activity in BeWo cells (Fig. 4C). In these experiments, another cAMP-inducing agent, SP-cAMP, was tested in parallel and again revealed induction of luciferase activity, as opposed to the inactive molecule RP-cAMP. Furthermore, the M1 mutant also led to the loss of this induction in transfected BeWo cells. To further support reduced promoter activation by the M1 mutation in the CRE/AP-1 motif, constructs were transfected in primary cytotrophoblasts and assessed for luciferase activity at day 2 posttransfection. Reporter gene expression indicated that the wild-type inducible region showed increased promoter activity while the mutant version of the construct demonstrated lower activity indicating the importance of the CRE/AP-1 motif for Syncytin-2 expression in cultured primary villous cytotrophoblasts (Fig. 4D).

As different transcription factors might bind to this CRE/AP-1 motif, we have concentrated our efforts in detecting interaction with JunD, CREB and CREB2. JunD was specifically chosen as it is the only Jun family member significantly expressed in human syncytiotrophoblast [43], and CREB and CREB2 having been previously implicated in the regulation of trophoblast genes [22, 34, 35] were also included. We first analyzed the binding of these factors to the CRE/AP-1 motif by supershift assays. Results were not conclusive (data not shown) and we thus switched to the more representative Chromatin immunoprecipitation approach (Fig. 4E). Compared to non-specific IgG, we indeed demonstrate that an *in vivo* specific interaction occurred between CREB, CREB2 and JunD on the Syncytin-2 promoter region encompassing the CRE/AP-1-binding site and that this interaction seemed more important for CREB2 and JunD, especially in forskolin-stimulated conditions.

These results thereby provided evidence that the CRE/AP-1-like motif was indeed critical for cAMP-induced modulation of Syncytin-2 expression and that the adjacent GATA-like binding site was less importantly implicated. Results further suggested that the CRE/AP-1-like motif in the Syncytin-2 promoter bound CREB, CREB2 and JunD.

### CREB/ATF family members are important for activation of the Syncytin-2 promoter and expression

**Fig 5:**
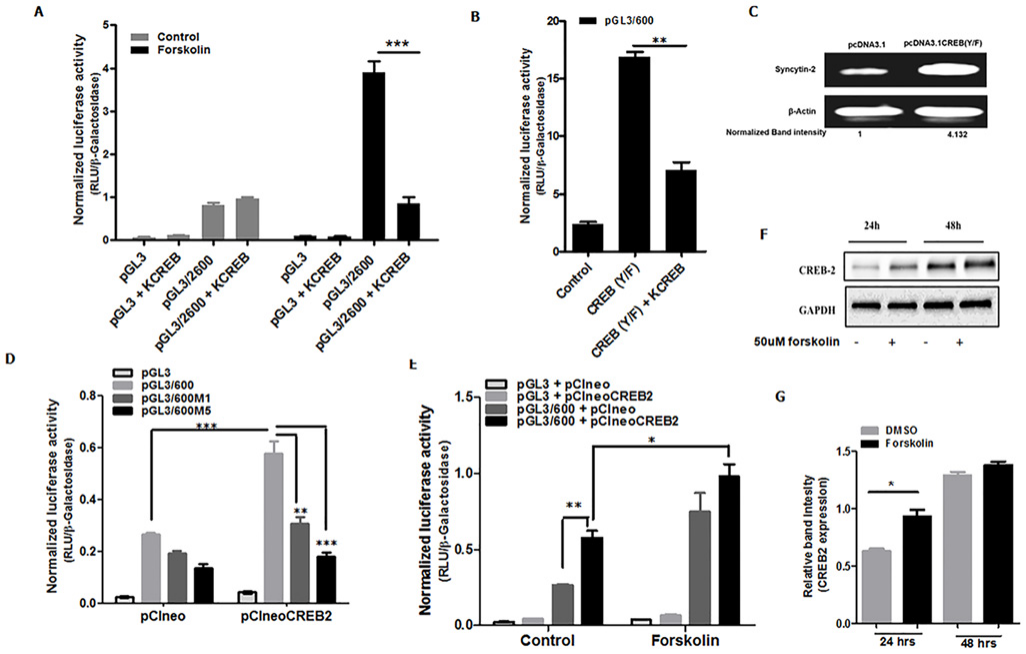
CREB2 induces activation of the Syncytin-2 promoter. **(A)** BeWo cells were co-transfected with pGL3/2600 (200 ng) (or pGL3basic), KCREB, a CREB dominant-negative mutant expression vector (vs. control vector) (200 ng) and pRcActin-LacZ (200 ng). At 36 h post transfection, cells were either left untreated (Control) or treated with forskolin. After 12 h of treatment, cells were harvested and analyzed for luciferase activity in triplicates. Luciferase activities were normalized over β-galactosidase activity and are expressed as the mean normalized RLU ± S.E.M. from three independently transfected cells in the same experiment. Results are representative of three independent experiments. **(B)** BeWo cells were co-transfected with pGL3/600 (200 ng), the constitutively activated CREB (Y/F) expression vector (200 ng), the CREB dominant-negative mutant expression vector (200 ng) (vs. control vector) and pRcActin-LacZ (200 ng). At 48 h post-transfection, cells were harvested and assayed for luciferase activity. Luciferase activities were normalized over β-galactosidase activity as the mean ± S.E.M. from three independently transfected cells in the same experiment. Results are representative of three independent experiments. **(C)** BeWo cells were transfected with the CREB (Y/F) expression vector (vs. the control empty vector) (200 ng). At 48 h post-transfection, total RNA were extracted and analyzed by RT-PCR for both Syncytin-2 and β-actin mRNA. Band intensities of Syncytin-2 mRNA after normalization with β-actin mRNA levels are indicated below with values for pcDNA3.1-transfected cells set as 1. (**D-E**) BeWo cells were co-transfected with pGL3/600 (400 ng) or equivalent constructs bearing the M1 or M5 mutant (**D**) along with pCIneoCREB2 (or the empty vector pCIneo) and pRcActin-LacZ (200 ng). In (**E**), cells were either left untreated or treated with forskolin prior to lysis. Luciferase activities were normalized over β-galactosidase activity as the mean ± S.E.M. from three independently transfected cells in the same experiment. Results are representative of two independent experiments. (**F-G**) BeWo cells were treated or not with forskolin for 24 and 48 h and cell extracts were subsequently analyzed for CREB2 and GAPDH protein levels by Western blot. Band intensities for CREB2 are depicted in (**G**) following normalization with GAPDH signals and were calculated as the mean ratio ± S.E.M. from three independent experiments (*p< 0.05; **p< 0.01; ***p< 0.001).

Previous studies have indicated that CREB could act as homodimers or heterodimers, involving Jun family members [44], which suggest that both factors could act as an heterodimer on the Syncytin-2 promoter. We first addressed the potential implication of CREB transcription factors in the modulation of Syncytin-2 promoter activity. We thus tested whether a general dominant-negative mutant of CREB (KCREB), capable of blocking several CREB family members by direct protein binding, could negatively impact the induction of the Syncytin-2 promoter following forskolin treatment. BeWo cells were hence transfected with the KCREB expression vector along with pGL3/2600. As expected, forskolin treatment augmented promoter activity, while the expression of KCREB completely inhibited its induced activity (Fig. 5A). To further confirm the role of CREB family members in Syncytin-2 promoter activation, we next tested a constitutively active version of CREB, in which tyrosine 134 is mutated for a phenylalanine [41]. Upon expression in BeWo cells, promoter activity from the pGL3/600 construct was increased by 18 fold over the empty control vector (Fig. 5B). This induction was notably reduced when BeWo cells were co-transfected with the KCREB expression vector. Furthermore, BeWo cells transfected with the constitutively activated CREB expression vector demonstrated a marked increase in Syncytin-2 mRNA level upon RT-PCR, analysis (Fig. 5C).

We next addressed the involvement of CREB2 in the modulation of the Syncytin-2 promoter. This CREB member was chosen as it demonstrated higher affinity to the CRE/AP-1 site in comparison to CREB. BeWo cells were hence transfected with a CREB2 expression vector, and as expected, CREB2 expression increased the activity of the-600 to +51 promoter region (pGL3/600) (Fig. 5D). Furthermore, when CREB2-induced promoter activity was compared between wild-type vs. M1 and M5-mutated constructs (pGL3/600M1 and pGL3/600M5), promoter activation was significantly reduced in mutant M1 and M5. Again, reduction in basal promoter activity was noticed, mostly in mutant M5, suggesting that the mutation in the CRE/AP-1-like motif overlapping the potential GATA-binding site was affecting basal activity. Although this induction was further enhanced when cells were treated with forskolin, a more modest increase was noted when compared to forskolin-treated cells, which were not expressing CREB2 (Fig. 5E). To determine if a forskolin-dependent increase in CREB2 expression paralleled induced Syncytin-2 expression, Western blot analyses was performed on BeWo cell extracts and indicated that forskolin induced endogenous CREB2 expression after 24 h of treatment, although no difference in CREB2 expression was observed at 48 h (Fig. 5F-G). Surprisingly, no such differences between untreated and forskolin-treated cells were noted at the mRNA level (data not shown).

These results hence indicated that CREB2 is implicated in the positive modulation of Syncytin-2 promoter activity upon forskolin treatment.

### JunD increases both Syncytin-2 promoter activity and expression

**Fig 6:**
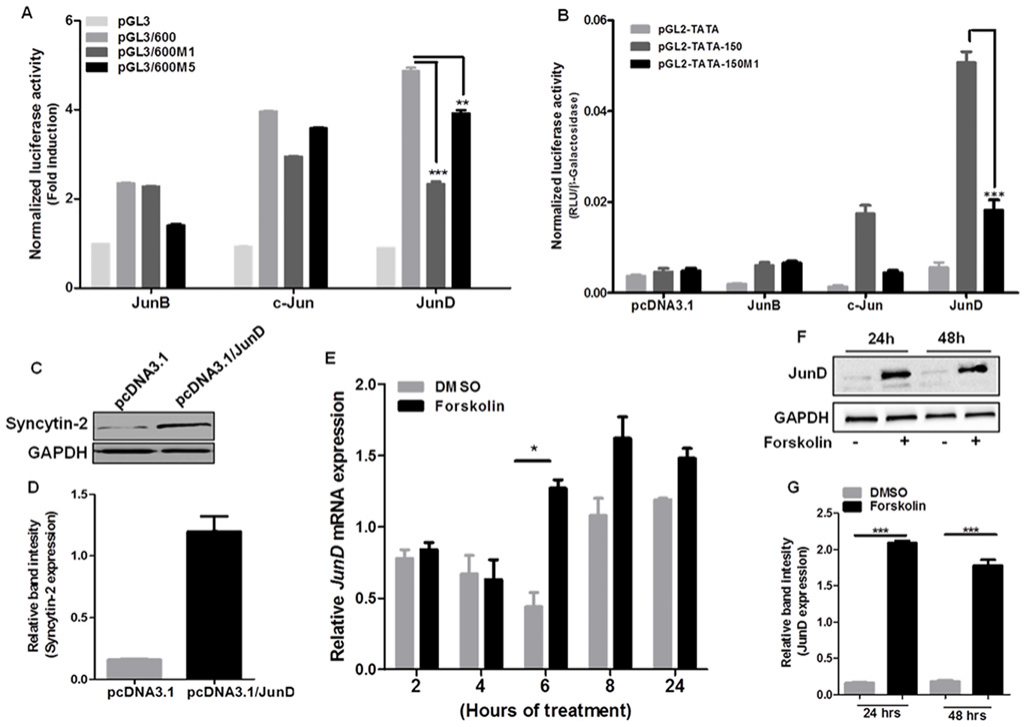
Modulation of Syncytin-2 promoter activity by various Jun family members. (**A**) JunB, c-Jun and JunD expression vectors (vs. control vector pcDNA3.1) (200 ng) were co-transfected with pGL3/600, pGL3/600M1, pGL3/600M5 or pGL3basic (200 ng) along with pRcActin-LacZ (200 ng) in BeWo cells. **(B)**. JunB, c-Jun and JunD expression vectors (vs. control vector pcDNA3.1) (200 ng) were co-transfected with pGL2-TATA-150, pGL2-TATA-150M1 or pGL2-TATA (200 ng) along with pRcActin-LacZ (200 ng) in BeWo cells. At 48 h post-transfection, cells were harvested and assayed for luciferase activity. Luciferase activities were normalized for β-galactosidase activity and are expressed as the mean normalized RLU ±S.E.M of three independent transfections for panel **B**. In panel **A**, mean fold induction values were determined by calculating normalized luciferase activities over values from corresponding control vector-transfected cells. Results are representative of three independent experiments. (**C-D**) BeWo cells were treated or not with forskolin for 24 h and cell extracts were subsequently analyzed for Syncytin-2 and GAPDH protein levels by Western blot. Band intensities for Syncytin-2 are depicted in (**D**) following normalization with GAPDH signals and were calculated as the mean ratio ± S.E.M. from three independent experiments. (**E-G**) BeWo cells were stimulated or not with forskolin for 48 h from which RNA (**E**) and proteins (**F-G**) were subsequently isolated. RT-PCR analyses were conducted and amplified JunD cDNAs were quantified for each time point following normalization with amplified β-actin mRNA (**E**), For Western blot analyses, anti-JunD and anti-GAPDH antibodies were used and JunD protein levels normalized for GAPDH were calculated and compared (**G**) (*p< 0.05; **p< 0.01; ***p< 0.001).

To assess the importance of JunD in the induction of Syncytin-2 promoter activity, co-transfection experiments were conducted with pGL3/600, pGL3/600M1 and pGL3/600M5 and expression vectors for various Jun family members (Fig. 6A). Upon co-transfection of BeWo cells, Syncytin-2 promoter activity was most significantly induced when JunD was overexpressed, although c-Jun expression was also mediating near comparable induction of the promoter. Both tested promoter mutants showed a significantly lesser response toward JunD overexpression solely. A similar trend was observed for promoter activation in overexpression condition for both JunB and c-Jun, although mutants had a lesser effect over their transactivation potential on the Syncytin-2 promoter. When expression of all Jun members was tested in BeWo cells along with WT or M1-mutated pGL2-TATA-150 constructs, JunD, as compared to other Jun members, again maximally increased the activity of the 150 bp fragment of the promoter (Fig. 6B). This induction was dramatically reduced in pGL2-TATA-150M1-transfected cells. Importantly, overexpression of JunD did not only increase Syncytin-2 promoter activity but also induced Syncytin-2 expression, as determined by Western blot analyses of transfected cells (Fig. 6C-D). To specifically investigate the effect of forskolin on JunD expression, BeWo cells were stimulated with forskolin in a time-dependent manner and JunD transcript and protein levels were evaluated. At 6 h post-stimulation, JunD mRNA were significantly increased (Fig. 6E). In addition, JunD protein levels were importantly induced at both 24 and 48 h of stimulation (Fig. 6F-G).

These results showed that JunD is an important regulator of Syncytin-2 in BeWo cells and its induction capacity was mediated via the CRE/AP-1-like motif. Furthermore, forskolin treatment of BeWo cells led to an important increase in JunD expression levels.

### CREB2 and JunD expression increases BeWo cell fusion

**Fig 7:**
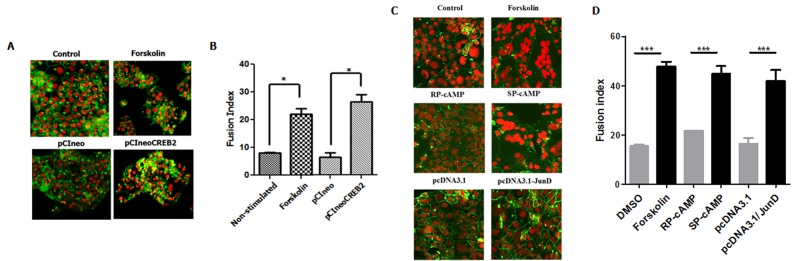
CREB2 and JunD overexpression induce BeWo fusion. BeWo cells were either left untreated (Control) or stimulated with forskolin or SP-cAMP (vs. its control, RP-cAMP) (**C-D**). In parallel, BeWo cells were transfected with expression vectors for JunD or CREB2 (or corresponding empty vectors) (200 ng). At 48 h post-transfection or post-stimulation, cell fusion was analyzed with anti-desmoplakin antibodies for membrane staining (green) and propidium iodide for nuclei staining (red). Images were visualized by confocal microscopy. **(B, D)** Cell fusion index was calculated in stimulated or transfected cells as follow: a total of 200 nuclei were counted in five independent fields per condition (average total of 1000 nuclei) and a percentage was calculated for the number of nuclei comprised in syncytia. These results are representative of a three different experiments (*p< 0.05; ***p< 0.001).

We next investigated whether CREB2 and JunD expression could induce BeWo cell fusion. BeWo cells were thus transfected with the CREB2 or JunD expression vectors (vs. transfected empty vectors) and then assessed for cell fusion (Fig. 7). In these experiments, forskolin and SP-cAMP treatment were used as positive controls, while negative controls consisted of untreated and RP-cAMP-treated cells. Cell fusion was assessed by staining of membranes with anti-desmoplakin antibodies and nuclear staining with propidium iodide. As expected forskolin and SP-cAMP treatment led to cell fusion, while basal cell fusion was measured in untreated, RP-cAMP-treated and empty vector-transfected cells. Importantly, CREB2 and JunD expression markedly induced cell fusion.

These results hence strongly suggested that CREB2 and JunD mediated BeWo cell fusion through induced Syncytin-2 expression.

### Knockdown of CREB2 and JunD expression reduces cell fusion and concomitant Syncytin-2 expression

**Fig 8:**
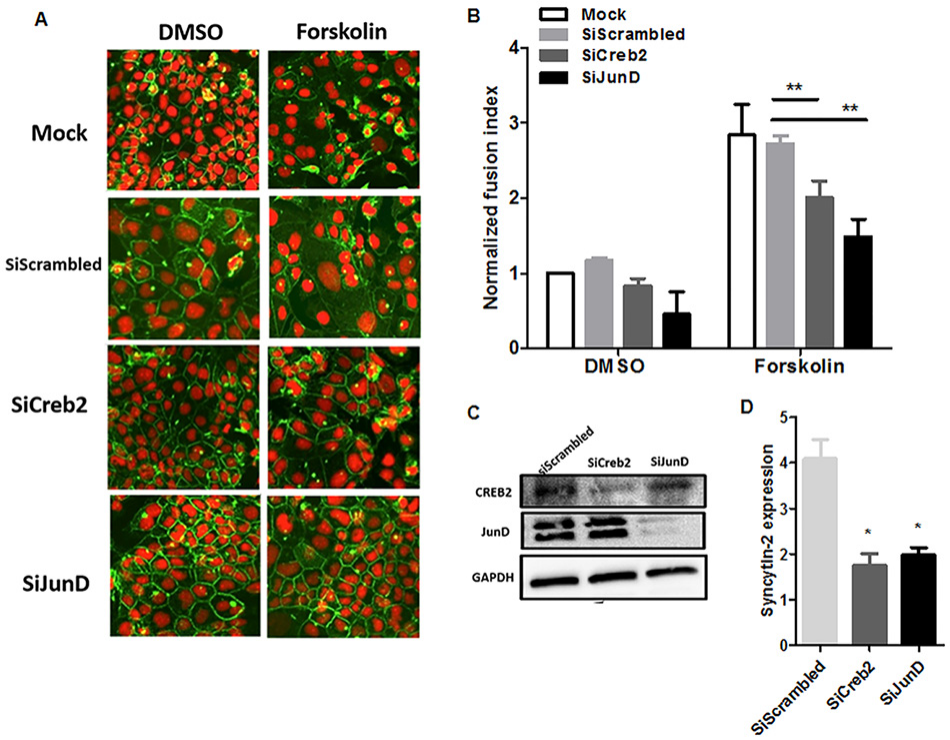
Silencing of either JunD or CREB2 reduces Syncytin-2 expression and fusion of stimulated BeWo cells. BeWo cells were stimulated with forskolin or left untreated (DMSO) and transfected with 37,5 ng CREB-2- or JunD-specific siRNA vs. scrambled control siRNA. (**A, B**) Cell fusion index was calculated for each transfected cell samples by confocal microscopy analyzed with anti-desmoplakin antibodies and propidium iodide staining. (**C-D**) Western blot analyses were performed on cellular extracts from each transfected cell samples using anti-JunD, anti-CREB2 and anti-GAPDH antibodies (left panel). Analyses for Syncytin-2 proteins levels were normalized against GAPDH signals and band intensities are depicted in (**D**) (*p< 0.05; **p< 0.01).

To further confirm the significance of CREB2 and JunD expression on cell fusion, BeWo cells were transfected with siRNA specific for either CREB2 or JunD mRNAs vs. scrambled siRNAs, as a negative control. After stimulation with forskolin, cell fusion was measured. Fusion events were significantly reduced in BeWo cells when CREB2 or JunD expression was silenced (Fig. 8A-B), while the control siRNA demonstrated little effect on cell fusion. Specific knockdown of CREB2 and JunD expression was confirmed by Western blot analyses of extracts derived from transfected cells and further indicated concomitant reduced Syncytin-2 protein levels (Fig. 8C-D).

These results hence confirmed that both CREB2 and JunD are important regulators of cell fusion and Syncytin-2 expression.

## Discussion

The ability of villous cytotrophoblasts to fuse and form the syncytiotrophoblast layer is a critical step during the development of the placenta. Two fusogenic proteins, Syncytin-1 and Syncytin-2 embedded in ancient proviral ERVW-1 and ERVFRD-1, respectively have been identified to play a major role in trophoblast fusion [6–10, 25–30]. The Syncytin-1 promoter has been well characterized and former studies have revealed that different transcription factors, including GATA2 and GATA3, are essential for the regulation of Syncytin-1 expression [24, 45, 46]. However, the placenta-specific transcription factor GCM1 and its targeted promoter region was demonstrated to be determinant in the regulation of Syncytin-1 expression and thereby fusion of trophoblasts [23, 47, 48]. Other studies have also shown that CpG methylation was important for the regulation of Syncytin-1 expression and varied in a stage-specific manner of pregnancy [49, 50]. On the other hand, little is known as to the regulation of basal levels or induced Syncytin-2 expression. The aim of the current study was thereby to characterize the Syncytin-2 promoter and to identify transcription factors involved in its regulation. We show that an important CRE/AP-1-like element located at position-203/-197 is involved in induced Syncytin-2 expression and that binding of CREB2 and JunD is likely mediating its modulatory potential.

In the present study, we have used the trophoblast-like choriocarcinoma BeWo cell line as a model for trophoblast fusion. Upon forskolin stimulation, BeWo cell fusion occurs with a concomitant increase in Syncytin-2 expression [29, 30]. The Syncytin-2 5’ flanking region from position-2600 was thus cloned, progressively deleted and first tested in BeWo cells stimulated with forskolin. These analyses revealed the presence of a maximal forskolin-inducible region consisting of the first 450 bp within the 5’flanking region upstream of the previously identified transcription initiation site [30]. Our results confirmed that, in BeWo cells, Syncytin-2 promoter activity might also depend on GCM1 upon induction, as deletion of the-450/-300 region containing the predicted GCM1-binding site led to a reproducible, albeit partial loss of forskolin responsiveness. However, our results argued that a more important region located between position-300 and-150 was essential for forskolin-mediated promoter induction. Indeed, when the promoter region was deleted up to position-150, forskolin-induced promoter activity was completely abolished, which suggested the presence of important DNA-binding sites within the-300 to-150 bp region. Further evidence for its importance in the regulation of Syncytin-2 promoter activity in BeWo cells was provided by experiments using this fragment in the context of a minimal promoter (a TATA box). Our results also demonstrated that the 150 bp forskolin-responsive Syncytin-2 promoter region was poorly induced in the non-trophoblastic 293T cell line. This might be reminiscent of previous observations from Liang *et al*. (2010), which have suggested that Syncytin-2 expression was strongly hampered by hypermethylation of the promoter region in non-trophoblastic cells such as MCF-7 and 293T [33].

In subsequent analyses, we have identified an enhancer in the Syncytin-2 promoter region, which showed high responsiveness toward forskolin. Data from EMSA assays demonstrated the formation of two different complexes binding to sequences located between position-211 to-177 nucleotides containing a CRE/AP-1-like motif and a potential GATA-binding site. Each specific complex was shown to bind to either the CRE/AP-1-like motif or the potential GATA-binding site. Mutation of the CRE/AP-1-like motif specifically weakened binding of the upper complex. Mutating this motif in the context of the forskolin-responsive region (150 bp) or the 600 bp promoter region equally reduced their responsiveness toward forskolin. The M6 mutation, which specifically affected binding of the lower complex, also corresponded to modification of the presumed GATA-binding site. However, no effect was seen on basal induced or forskolin-induced promoter activity upon specific mutation of this region in the promoter. However, this GATA-binding site, which we presumed to interact with the lower complex, might nonetheless play a role in promoter activity in a different cell context. For example, GATA2 and GATA3 are transcription factors involved in Syncytin-1 gene regulation [24] and their mutual binding has been suggested to be important for regulation of the expression of trophoblast-specific genes [51]. It is possible that complexes bound to the CRE/AP-1-like motif could be competed by GATA binding factors, which could thereby contribute to an exclusive modulation of Syncytin-2 expression in different conditions. This possibility is in line with our EMSA results demonstrating that both factors bind at close proximity and in fact share certain nucleotides for proper binding, as judged by competition experiments with oligonucleotide M5. Furthermore, experiments with the M5 mutated promoter constructs suggest that this element could be involved in basal promoter activity. Further analyses are needed to address these issues and to clearly identify the cellular factor binding to this region of the Syncytin-2 promoter.

Given the drastic impact of the mutated CRE/AP-1 motif on Syncytin-2 promoter activity, we have thus focused on identifying proteins, which bound to this region. Based on ChIP experiments, our data suggest that CREB2 (ATF4) (and likely CREB) and JunD bound the CRE/AP-1-like motif. This is also supported by overexpression studies in which both transcription factors positively modulated Syncytin-2 promoter activity in a CRE/AP-1-like motif-dependent manner. Furthermore, when constitutively active CREB was overexpressed in non-stimulated BeWo cells, Syncytin-2 mRNA levels was increased. It is known that CREB2 and other CREB family members can activate LTRs of different exogenous retroviruses such as HIV-1 [52, 53] and HTLV-1 [42, 54]. It is thus conceivable that members of this family of transcription factors are also involved in the modulation of promoters from ERVs such as the Syncytin-2-containing ERVFRD-1. However, although CREB/ATF family members are generally known to be induced by the cAMP-dependent PKA signaling pathway [55, 56], CREB2 appears to lack a PKA phosphorylation site, although CREB2 phosphorylation has been nonetheless formerly described [57]. We have however observed that CREB2 levels slightly increases upon forskolin stimulation, which could in this case contribute to increased modulation of Syncytin-2 expression. More studies are needed to precisely determine its implication (or that of other CREB/ATF family members) in the modulation of Syncytin-2 expression.

As a basic leucine zipper (bZIP)-containing transcription factor, CREB2/ATF4) either homodimerizes or heterodimerizes with other bZIP transcription factors, such as Jun family members, which typically bind to binding sites sharing a high similarity with CRE motifs [36, 37]. Our results indeed indicate that JunD could be part of the complex binding to the CRE/AP-1-like motif. Testing of other Jun family members by transfection experiments revealed that other Jun family members were not as efficient in increasing Syncytin-2 promoter activity, although our results also pointed to possible involvement of c-Jun. However, since a previous study had revealed that JunD is the only Jun family member expressed in the syncytiotrophoblast [43], we are more confident that JunD is the determinant member in the regulation of Syncytin-2 in BeWo cells and primary trophoblasts and in fact, we have observed an important increase in JunD protein levels upon forskolin treatment of BeWo cells. Our results also agree with our previous publication showing that Syncytin-2 expression increased significantly in primary cytotrophoblasts at day 3 and 4 of culture, when these cells fuse to form a syncytiotrophoblast-like layer [30]. On the other hand, *in vivo* studies have revealed that the Syncytin-2 protein is instead restricted to villous cytotrophoblasts [27]. No clear explanation can be provided over this discrepancy and more studies will be needed to address this issue.

**Fig 9:**
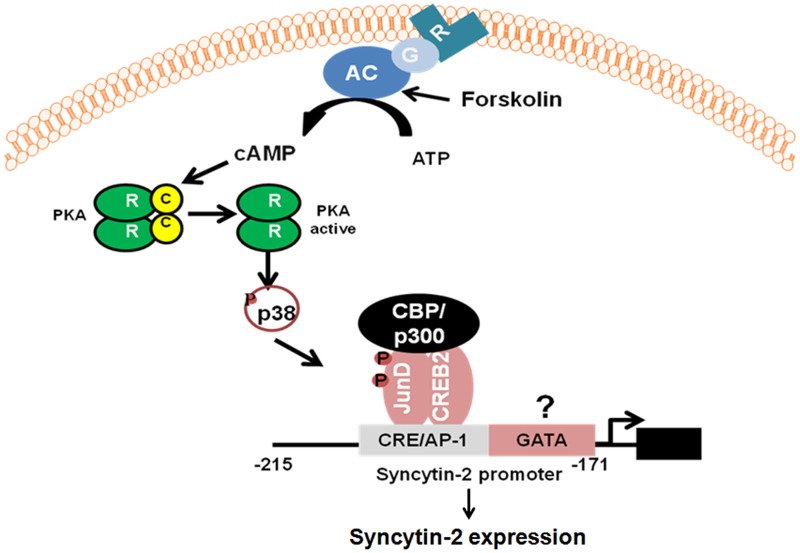
Proposed model for CREB2/JunD-mediated activation of Syncytin-2 expression. Based on the experiments shown in this study, we propose that p38MAPK activation upon forskolin stimulation activates both CREB2 and JunD. These transcription factors bind to the CRE/AP-1 motif, possibly as an heterodimer. The specific binding of this complex to the Syncytin-2 promoter then recruits CBP/p300, which mediates histone acetylation and transcription. An unknown factor binds next to this region at a presumed GATA-binding site and might compete for binding, thereby acting negatively on CREB2/JunD-induced Syncytin-2 transcription. Possible alternatives for this model include the interaction of other CREB/ATF family members to the CRE/AP-1 motif.

Our results have further demonstrated that CREB2 and JunD overexpression in non-stimulated BeWo cells led to cell fusion. In contrast, repression of either gene in stimulated BeWo cells reduced cell fusion. Similarly to GCM1 [58], CREB2 and JunD are thus additional transcription factors which, upon induced expression, results in BeWo cell fusion. Interestingly, it has been reported that CREB/ATF family members are also active players in the regulation of GCM1 expression [59], thereby suggesting that forskolin induction of various CREB/ATF family members could be essential for inducing expression of different genes, such as Syncytin-2, mediating trophoblast fusion. Based on the above results, we propose a model illustrating the possible mechanism of regulation of Syncytin-2 expression by a CREB2/JunD heterodimer (Fig. 9). As previous studies have identified pathways by which fusogenic genes can be modulated following forskolin stimulation, such as MAPK14 and PKA [22, 60], we suggest that an initial increase in cAMP levels leads to activation of different serine/threonine kinases, which include PKA activation leading to MAPK14 phosphorylation. These kinases then phosphorylate CREB/ATF family members causing induced expression of several trophoblast-specific genes, including Syncytin-2. The activation of the Syncytin-2 promoter is also likely PKA-independent and might implicate an additional pathway, which would induce JunD expression with potential phosphorylation. The binding of CREB2/JunD heterodimers to the CRE/AP-1-like motif would subsequently lead to the binding of CBP or p300 and concomitant acetylation of histones. In this model, the binding of the presumed GATA factor might also contribute in the regulation of the Syncytin-2 promoter in a different context.

## Conclusions

In summary, our findings provide important evidence for the implication of CREB and Jun transcription factors in cAMP-mediated induction of Syncytin-2 expression. More specifically, our results suggest that CREB2 and JunD form an heterodimer on a CRE/AP-1-like motif and strongly upregulate the expression of Syncytin-2 thereby leading to trophoblast fusion. The implication of a member of the Jun and CREB family in the modulation of Syncytin-2 expression may represent a convergence between various signaling cascades that may equally be important in regulation of the expression of other genes essential for placenta development.

## References

[1] Muyan M, Boime I. Secretion of chorionic gonadotropin from human trophoblasts. Placenta 1997;18:237–241. 9179915 10.1016/s0143-4004(97)80056-2

[2] Morrish DW, Dakour J, Li H. Functional regulation of human trophoblast differentiation. J Reprod Immunol 1998;39:179–195. 9786461 10.1016/s0165-0378(98)00021-7

[3] Handwerger S, Freemark M. The roles of placental growth hormone and placental lactogen in the regulation of human fetal growth and development. J Pediatr Endocrinol Metab 2000;13:343–356. 10776988 10.1515/jpem.2000.13.4.343

[4] Lacroix MC, Guibourdenche J, Frendo JL, Pidoux G, Evain-Brion D. Placental growth hormones. Endocrine 2002;19:73–79. 12583604 10.1385/ENDO:19:1:73

[5] Uzan J, Carbonnel M, Piconne O, Asmar R, Ayoubi JM. Pre-eclampsia: pathophysiology, diagnosis, and management. Vasc Health Risk Manag 2011;7:467–474. 10.2147/VHRM.S20181 21822394 PMC3148420

[6] Frendo JL, Olivier D, Cheynet V, Blond JL, Bouton O, Vidaud M, et al Direct involvement of HERV-W Env glycoprotein in human trophoblast cell fusion and differentiation. Mol Cell Biol 2003;23:3566–3574. 12724415 10.1128/MCB.23.10.3566-3574.2003 PMC164757

[7] Mi S, Lee X, Li X, Veldman GM, Finnerty H, Racie L, et al Syncytin is a captive retroviral envelope protein involved in human placental morphogenesis. Nature 2000;403:785–789. 10693809 10.1038/35001608

[8] Blond JL, Lavillette D, Cheynet V, Bouton O, Oriol G, Chapel-Fernandes S, et al An envelope glycoprotein of the human endogenous retrovirus HERV-W is expressed in the human placenta and fuses cells expressing the type D mammalian retrovirus receptor. J Virol 2000;74:3321–3329. 10708449 10.1128/jvi.74.7.3321-3329.2000 PMC111833

[9] Blond J-L, Besème F, Duret L, Bouton O, Bedin F, Perron H, et al Molecular characterization and placental expression of HERV-W, a new human endogenous retrovirus family. J. Virol. 1999;73:1175–1185. 9882319 10.1128/jvi.73.2.1175-1185.1999 PMC103938

[10] Kudo Y, Boyd CA. Changes in expression and function of syncytin and its receptor, amino acid transport system B(0) (ASCT2), in human placental choriocarcinoma BeWo cells during syncytialization. Placenta 2002;23:536–541. 12175968 10.1053/plac.2002.0839

[11] Marin M, Lavillette D, Kelly SM, Kabat D. N-linked glycosylation and sequence changes in a critical negative control region of the ASCT1 and ASCT2 neutral amino acid transporters determine their retroviral receptor functions. J Virol 2003;77:2936–2945. 12584318 10.1128/JVI.77.5.2936-2945.2003 PMC149750

[12] Lee X, Keith JC Jr., Stumm N, Moutsatsos I, McCoy JM, Crum CP, et al Downregulation of placental syncytin expression and abnormal protein localization in pre-eclampsia. Placenta 2001;22:808–812. 11718567 10.1053/plac.2001.0722

[13] Chen CP, Wang KG, Chen CY, Yu C, Chuang HC, Chen H. Altered placental syncytin and its receptor ASCT2 expression in placental development and pre-eclampsia. Bjog 2006;113:152–158. 16411991 10.1111/j.1471-0528.2005.00843.x

[14] Keith JC Jr., Pijnenborg R, Van Assche FA. Placental syncytin expression in normal and preeclamptic pregnancies. Am J Obstet Gynecol 2002;187:1122–1123; author reply 1123–1124. 12389018 10.1067/mob.2002.128512

[15] Knerr I, Beinder E, Rascher W. Syncytin, a novel human endogenous retroviral gene in human placenta: evidence for its dysregulation in preeclampsia and HELLP syndrome. Am J Obstet Gynecol 2002;186:210–213. 11854637 10.1067/mob.2002.119636

[16] Knerr I, Huppertz B, Weigel C, Dotsch J, Wich C, Schild RL, et al Endogenous retroviral syncytin: compilation of experimental research on syncytin and its possible role in normal and disturbed human placentogenesis. Mol Hum Reprod 2004;10:581–588. 15181178 10.1093/molehr/gah070

[17] Kudaka W, Oda T, Jinno Y, Yoshimi N, Aoki Y. Cellular localization of placenta-specific human endogenous retrovirus (HERV) transcripts and their possible implication in pregnancy-induced hypertension. Placenta 2008;29:282–289. 18158183 10.1016/j.placenta.2007.11.009

[18] Langbein M, Strick R, Strissel PL, Vogt N, Parsch H, Beckmann MW, et al Impaired cytotrophoblast cell-cell fusion is associated with reduced Syncytin and increased apoptosis in patients with placental dysfunction. Mol Reprod Dev 2008;75:175–183. 17546632 10.1002/mrd.20729

[19] Vargas A, Toufaily C, LeBellego F, Rassart E, Lafond J, Barbeau B. Reduced expression of both syncytin 1 and syncytin 2 correlates with severity of preeclampsia. Reprod Sci 2011;18:1085–1091. 10.1177/1933719111404608 21493955

[20] Knerr I, Weigel C, Linnemann K, Dotsch J, Meissner U, Fusch C, et al Transcriptional effects of hypoxia on fusiogenic syncytin and its receptor ASCT2 in human cytotrophoblast BeWo cells and in ex vivo perfused placental cotyledons. Am J Obstet Gynecol 2003;189:583–588. 14520239 10.1067/s0002-9378(03)00538-6

[21] Zhuang XW, Li J, Brost BC, Xia XY, Chen HB, Wang CX, et al Decreased expression and altered methylation of syncytin-1 gene in human placentas associated with preeclampsia. Curr Pharm Des 2014;20:1796–1802. 23888950 10.2174/13816128113199990541

[22] Delidaki M, Gu M, Hein A, Vatish M, Grammatopoulos DK. Interplay of cAMP and MAPK pathways in hCG secretion and fusogenic gene expression in a trophoblast cell line. Mol Cell Endocrinol 2011;332:213–220. 10.1016/j.mce.2010.10.013 21035520

[23] Yu C, Shen K, Lin M, Chen P, Lin C, Chang GD, et al GCMa regulates the syncytin-mediated trophoblastic fusion. J Biol Chem 2002;277:50062–50068. 12397062 10.1074/jbc.M209316200

[24] Cheng YH, Handwerger S. A placenta-specific enhancer of the human syncytin gene. Biol Reprod 2005;73:500–509. 15888734 10.1095/biolreprod.105.039941

[25] Blaise S, de Parseval N, Benit L, Heidmann T. Genomewide screening for fusogenic human endogenous retrovirus envelopes identifies syncytin 2, a gene conserved on primate evolution. Proc Natl Acad Sci U S A 2003;100:13013–13018. 14557543 10.1073/pnas.2132646100 PMC240736

[26] Blaise S, Ruggieri A, Dewannieux M, Cosset FL, Heidmann T. Identification of an envelope protein from the FRD family of human endogenous retroviruses (HERV-FRD) conferring infectivity and functional conservation among simians. J Virol 2004;78:1050–1054. 14694139 10.1128/JVI.78.2.1050-1054.2004 PMC368808

[27] Malassine A, Blaise S, Handschuh K, Lalucque H, Dupressoir A, Evain-Brion D, et al Expression of the fusogenic HERV-FRD Env glycoprotein (syncytin 2) in human placenta is restricted to villous cytotrophoblastic cells. Placenta 2007;28:185–191. 16714059 10.1016/j.placenta.2006.03.001

[28] Malassine A, Frendo JL, Blaise S, Handschuh K, Gerbaud P, Tsatsaris V, et al Human endogenous retrovirus-FRD envelope protein (syncytin 2) expression in normal and trisomy 21-affected placenta. Retrovirology 2008;5:6 10.1186/1742-4690-5-6 18215254 PMC2245979

[29] Chen CP, Chen LF, Yang SR, Chen CY, Ko CC, Chang GD, et al Functional characterization of the human placental fusogenic membrane protein syncytin 2. Biol Reprod 2008;79:815–823. 10.1095/biolreprod.108.069765 18650494

[30] Vargas A, Moreau J, Landry S, LeBellego F, Toufaily C, Rassart E, et al Syncytin-2 plays an important role in the fusion of human trophoblast cells. J Mol Biol 2009;392:301–318. 10.1016/j.jmb.2009.07.025 19616006

[31] Esnault C, Priet S, Ribet D, Vernochet C, Bruls T, Lavialle C, et al A placenta-specific receptor for the fusogenic, endogenous retrovirus-derived, human syncytin-2. Proc Natl Acad Sci U S A 2008;105:17532–17537. 10.1073/pnas.0807413105 18988732 PMC2582322

[32] Toufaily C, Vargas A, Lemire M, Lafond J, Rassart E, Barbeau B. MFSD2a, the Syncytin-2 receptor, is important for trophoblast fusion. Placenta 2013;34:85–88. 10.1016/j.placenta.2012.10.012 23177091

[33] Liang CY, Wang LJ, Chen CP, Chen LF, Chen YH, Chen H. GCM1 regulation of the expression of syncytin 2 and its cognate receptor MFSD2A in human placenta. Biol Reprod 2010;83:387–395. 10.1095/biolreprod.110.083915 20484742

[34] Schubert SW, Abendroth A, Kilian K, Vogler T, Mayr B, Knerr I, et al bZIP-Type transcription factors CREB and OASIS bind and stimulate the promoter of the mammalian transcription factor GCMa/Gcm1 in trophoblast cells. Nucleic Acids Res 2008;36:3834–3846. 10.1093/nar/gkn306 18495750 PMC2441803

[35] Zhou Z, Wang R, Yang X, Lu XY, Zhang Q, Wang YL, et al The cAMP-responsive element binding protein (CREB) transcription factor regulates furin expression during human trophoblast syncytialization. Placenta 2014;35:907–918. 10.1016/j.placenta.2014.07.017 25175744

[36] Chevray PM, Nathans D. Protein interaction cloning in yeast: identification of mammalian proteins that react with the leucine zipper of Jun. Proc Natl Acad Sci U S A 1992;89:5789–5793. 1631061 10.1073/pnas.89.13.5789 PMC402103

[37] Hai T, Curran T. Cross-family dimerization of transcription factors Fos/Jun and ATF/CREB alters DNA binding specificity. Proc Natl Acad Sci U S A 1991;88:3720–3724. 1827203 10.1073/pnas.88.9.3720 PMC51524

[38] Pattillo RA, Gey GO, Delfs E, Mattingly RF. Human hormone production in vitro. Science 1968;159:1467–1469. 5753554 10.1126/science.159.3822.1467

[39] Langlois M, Audet B, Legault E, Pare ME, Ouellet M, Roy J, et al Activation of HTLV-I gene transcription by protein tyrosine phosphatase inhibitors. Virology 2004;329:395–411. 15518818 10.1016/j.virol.2004.09.003

[40] Walton KM, Rehfuss RP, Chrivia JC, Lochner JE, Goodman RH. A dominant repressor of cyclic adenosine 3',5'-monophosphate (cAMP)-regulated enhancer-binding protein activity inhibits the cAMP-mediated induction of the somatostatin promoter in vivo. Mol Endocrinol 1992;6:647–655. 1350057 10.1210/mend.6.4.1350057

[41] Du K, Asahara H, Jhala US, Wagner BL, Montminy M. Characterization of a CREB gain-of-function mutant with constitutive transcriptional activity in vivo. Mol. Cell. Biol. 2000;20:4320–4327. 10825195 10.1128/mcb.20.12.4320-4327.2000 PMC85799

[42] Gachon F, Peleraux A, Thebault S, Dick J, Lemasson I, Devaux C, et al CREB-2, a cellular CRE-dependent transcription repressor, functions in association with Tax as an activator of the human T-cell leukemia virus type 1 promoter. J Virol 1998;72:8332–8337. 9733879 10.1128/jvi.72.10.8332-8337.1998 PMC110203

[43] Bamberger AM, Bamberger CM, Aupers S, Milde-Langosch K, Loning T, Makrigiannakis A. Expression pattern of the activating protein-1 family of transcription factors in the human placenta. Mol Hum Reprod 2004;10:223–228. 14985474 10.1093/molehr/gah011

[44] Ameri K, Harris, Adrian. Activating transcription factor 4. The International Journal of Biochemistry & Cell Biology 2008;40:14–21. 17466566 10.1016/j.biocel.2007.01.020

[45] Cheng YH, Richardson BD, Hubert MA, Handwerger S. Isolation and characterization of the human syncytin gene promoter. Biol Reprod 2004;70:694–701. 14613893 10.1095/biolreprod.103.023473

[46] Mameli G, Astone V, Khalili K, Serra C, Sawaya BE, Dolei A. Regulation of the syncytin-1 promoter in human astrocytes by multiple sclerosis-related cytokines. Virology 2007;362:120–130. 17258784 10.1016/j.virol.2006.12.019

[47] Prudhomme S, Oriol G, Mallet F. A retroviral promoter and a cellular enhancer define a bipartite element which controls env ERVWE1 placental expression. J Virol 2004;78:12157–12168. 15507602 10.1128/JVI.78.22.12157-12168.2004 PMC525085

[48] Chang CW, Chuang HC, Yu C, Yao TP, Chen H. Stimulation of GCMa transcriptional activity by cyclic AMP/protein kinase A signaling is attributed to CBP-mediated acetylation of GCMa. Molecular and cellular biology 2005;25:8401–8414. 16166624 10.1128/MCB.25.19.8401-8414.2005 PMC1265739

[49] Matouskova M, Blazkova J, Pajer P, Pavlicek A, Hejnar J. CpG methylation suppresses transcriptional activity of human syncytin-1 in non-placental tissues. Exp Cell Res 2006;312:1011–1020. 16427621 10.1016/j.yexcr.2005.12.010

[50] Gimenez J, Montgiraud C, Oriol G, Pichon JP, Ruel K, Tsatsaris V, et al Comparative methylation of ERVWE1/syncytin-1 and other human endogenous retrovirus LTRs in placenta tissues. DNA Res 2009;16:195–211. 10.1093/dnares/dsp011 19561344 PMC2725788

[51] Bai H, Sakurai T, Kim MS, Muroi Y, Ideta A, Aoyagi Y, et al Involvement of GATA transcription factors in the regulation of endogenous bovine interferon-tau gene transcription. Mol Reprod Dev 2009;76:1143–1152. 10.1002/mrd.21082 19598245

[52] Mu Y, Yu Y, Yue X, Musarat I, Gong R, Zhu C, et al The X protein of HBV induces HIV-1 long terminal repeat transcription by enhancing the binding of C/EBPbeta and CREB1/2 regulatory proteins to the long terminal repeat of HIV-1. Virus Res 2011;156:81–90. 10.1016/j.virusres.2011.01.001 21237225

[53] Caselli E, Benedetti S, Gentili V, Grigolato J, Di Luca D. Short Communication: Activating Transcription Factor 4 (ATF4) Promotes HIV Type 1 Activation. AIDS Res Hum Retroviruses 2012;28:907–912. 10.1089/AID.2011.0252 22050711

[54] Reddy TR, Tang H, Li X, Wong-Staal F. Functional interaction of the HTLV-1 transactivator Tax with activating transcription factor-4 (ATF4). Oncogene 1997;14:2785–2792. 9190894 10.1038/sj.onc.1201119

[55] Montminy MR, Bilezikjian LM. Binding of a nuclear protein to the cyclic-AMP response element of the somatostatin gene. Nature 1987;328:175–178. 2885756 10.1038/328175a0

[56] Gonzalez GA, Menzel P, Leonard J, Fischer WH, Montminy MR. Characterization of motifs which are critical for activity of the cyclic AMP-responsive transcription factor CREB. Mol Cell Biol 1991;11:1306–1312. 1671708 10.1128/mcb.11.3.1306-1312.1991 PMC369401

[57] Elefteriou F, Ahn JD, Takeda S, Starbuck M, Yang X, Liu X, et al Leptin regulation of bone resorption by the sympathetic nervous system and CART. Nature 2005;434:514–520. 15724149 10.1038/nature03398

[58] Baczyk D, Drewlo S, Proctor L, Dunk C, Lye S, Kingdom J. Glial cell missing-1 transcription factor is required for the differentiation of the human trophoblast. Cell Death Differ 2009;16:719–727. 10.1038/cdd.2009.1 19219068

[59] Knerr I, Schubert SW, Wich C, Amann K, Aigner T, Vogler T, et al Stimulation of GCMa and syncytin via cAMP mediated PKA signaling in human trophoblastic cells under normoxic and hypoxic conditions. FEBS Lett 2005;579:3991–3998. 16004993 10.1016/j.febslet.2005.06.029

[60] Daoud G, Amyot M, Rassart E, Masse A, Simoneau L, Lafond J. ERK1/2 and p38 regulate trophoblasts differentiation in human term placenta. J Physiol 2005;566:409–423. 15890698 10.1113/jphysiol.2005.089326 PMC1464762

